# Pomegranate disease diagnosis with severity estimation and treatment remedies using deep learning and RAG-based LLM

**DOI:** 10.1038/s41598-025-25160-8

**Published:** 2025-11-21

**Authors:** A. R. Revathi, A. Arockia Agash

**Affiliations:** School of Computer Science and Engineering, Vellore Institute of Technology–Chennai, Chennai, Tamil Nadu 600127 India

**Keywords:** Pomegranate disease detection, Convolutional neural networks, Transfer learning, Grad-CAM ++, Mahalanobis distance, Retrieval-augmented generation

## Abstract

Pomegranate cultivation faces significant challenges due to fruit diseases that significantly impact crop yield and farmer income. Traditional methods for disease detection are often slow and prone to errors, delaying timely intervention. This paper proposes a deep learning-based system for automatic, multi-class disease classification in pomegranates using transfer learning. A dataset comprising 5099 annotated images was used to train and evaluate several CNN models, including DenseNet121, EfficientNetB0V2, MobileNetV2, ResNet50, VGG16, and InceptionV3. DenseNet121 emerged as the top performer, achieving an accuracy of 99.35%. To enhance practical value, a novel Healthy-Based Deviation Scoring (HBDS) method was developed to estimate disease severity using Grad-CAM ++ for lesion localization and Mahalanobis distance-based scoring, followed by Gaussian Mixture Model clustering. The severity predictions of the system were verified against manually labeled images, and the system has shown superior accuracy compared to pixel-based methods. Also, a recommendation module was integrated using a retrieval-augmented language model, which provides disease-specific treatment suggestions based on the predicted severity. The complete pipeline is implemented as a user-friendly web application that delivers real-time diagnosis, severity estimation, and actionable treatment plans, which offer a practical and scalable solution for modern precision agriculture.

## Introduction

Pomegranates originated from Iran and Afghanistan, and they are currently cultivated throughout America, Europe, Africa, Australia, India, and the Middle East due to their economic and nutritional value. In India, pomegranates are a substantial crop^1^. Maharashtra grows the most, giving 70% to 80% of the country’s supply. Pomegranate farming is also vital in states like Andhra Pradesh, Gujarat, and Karnataka^2^. Key places in Maharashtra, such as Solapur, Sangli, and Nashik, along with a few locations in Karnataka and Andhra Pradesh, have the proper temperatures to grow high-quality food that will last a long time. They contain many essential nutrients, such as fibre, potassium, vitamins C, K, and B5, as well as potent antioxidants like anthocyanins and punicalagins, which can promote health and reduce inflammation^3^. Both fresh and processed types of the fruit are common because they can be used in many ways, including being turned into juice and oil. Although pomegranates can be grown in various temperatures, diseases that affect them at different stages of development reduce their yield and cost farmers a lot of money, particularly in India^4^.

Traditionally, identifying these diseases has relied on manual inspections by experts, an approach that is often slow, subjective, and prone to errors. As a result, treatments may be delayed, leading to more extensive crop damage and a higher reliance on chemical inputs^5^. Fortunately, the advancements in artificial intelligence and computer vision have paved the way for more efficient solutions in modern agriculture. Deep learning has emerged as a powerful alternative for automating plant health monitoring, particularly through models like CNNs (Convolutional Neural Networks)^6,7^. In this study, a deep learning-based system is introduced, explicitly designed for multi-class disease classification in pomegranate fruits. The model is built on a dataset of 5099 annotated images, which identifies five distinct disease categories^8^. With the help of Transfer Learning, fine-tuned pre-trained models like DenseNet121 and VGG19 have been used to significantly improve model performance while reducing training time and resource requirements^9,10^. To address common challenges like class imbalance, data augmentation techniques such as Canny edge detection and color jittering, along with class weighting and other regularization methods, have been used. These strategies enhance the generalization of the model capabilities and ensure better recognition of rare or underrepresented disease classes^11,12^. Beyond disease classification, this work presents a novel framework called Healthy-Based Deviation Scoring (HBDS) for estimating disease severity. HBDS combines Grad-CAM ++ for lesion localization and Mahalanobis distance for evaluating deviation from healthy samples and Gaussian Mixture Models (GMM) to assign severity levels into Low, Medium, or High categories^13,14^.

To translate the predictions into real-world action, a Retrieval-Augmented Generation (RAG) based LLM assistant has been incorporated into the pipeline. This assistant searches a carefully selected vector database using the Mistral Small 3.1 (24B) model to create contextualized therapy recommendations tailored to the identified condition and severity level^15,16^. Additional features like PDF report generation and a user-friendly chatbot interface further support farmers and agronomists in decision-making^17^. Drawing inspiration from the role of machine learning in environmental analytics, this work contributes a holistic, scalable, and intelligent system that minimizes crop loss, limits chemical overuse, and promotes sustainability in pomegranate farming^18,19^.

This paper is organized as follows: section "Related works" reviews the recent literature on deep learning techniques applied to plant disease detection and severity estimation. Section "Methodology" presents the overall methodology, which includes dataset preparation and preprocessing, classification using various CNN-based architectures and the proposed Healthy-Based Deviation Scoring (HBDS) framework for severity analysis. Section "Model evaluation and result discussions" discusses model evaluation, comparing classification performance and severity estimation accuracy. Section "Conclusion and future work" introduces the recommendation module built using a Retrieval-Augmented Generation (RAG) approach and outlines its integration into a user-friendly deployment interface. The final section concludes the work and outlines directions for future research.

## Related works

Deep learning methods have lately produced significant improvements in agricultural diagnostics, especially in automating the process of disease detection in fruits. CNNs have been thoroughly adopted among various architectures for their ability to learn hierarchical patterns from leaf and fruit images. By employing these models, diseases in crops such as pomegranates, mangoes, guavas, apples, grapes, and citrus fruits have been effectively identified. Using transfer learning, Naseer et al.^20^ created a model that effectively identified several pomegranate growth stages with insufficient data, therefore showing how pretrained models may be customized to specific crop phenology. In terms of disease severity, scientists have shifted their focus from binary classification to infection estimation. With few labelled data, Bedi et al.^13^ suggested PDSE-Lite, a lightweight framework using convolutional autoencoders and few-shot learning to assess plant disease severity. Likewise, Kundu et al.^14^ presented a CNN-based severity prediction model for maize, which could also project related crop loss, adding value for economic planning in areas prone to diseases. Hybrid deep learning models have shown exceptional promise, particularly when data availability is a limiting factor. Abuhayi et al.^21^ demonstrated that a hybrid CNN architecture, combining LeNet and ResNet, can achieve high accuracy in classifying pumpkin leaf diseases, while Pacal et al.^7^ illustrated the effectiveness of merging CNNs with Vision Transformers to boost grapevine disease recognition. Such hybridization underscores the trend toward combining model strengths for robust diagnostics across varying conditions. Ashurov et al.^22^ showed that integrating squeeze and excitation blocks with depth-wise CNNs and residual skip connections can achieve lightweight yet highly accurate plant disease detection, reporting 98% accuracy with real-time feasibility. Al-Gaashani et al.^23^ demonstrated the effectiveness of multi-scale convolutional pooling in capturing both fine-grained and global lesion patterns, achieving 98.7% accuracy for maize diseases while remaining efficient for agricultural deployment.

Texture-based feature extraction also plays a role in disease detection. Bezabh et al.^24^ used GLCM-based descriptors and SVM to classify chickpea leaf diseases with high precision. Within pomegranate-specific studies, Mane et al.^25^ proposed a CNN model that reached over 90% accuracy in detecting bacterial blight from infected fruit images, while Gupta et al.^8^ enhanced this by integrating Random Forest with CNNs to classify multiple pomegranate disease categories effectively. The breadth of machine learning approaches has been comprehensively reviewed by Bhargava et al.^3^, who compared SVM, ANN, and CNN models for grading and disease detection in fruits and vegetables. As model diversity has increased, the use of ensemble strategies and advanced classifiers has also increased, as noted by Yang et al.^26^. In that study, MobileNetV2 was combined with SVM to detect cherry disease, achieving a reported accuracy of 98.3%. Transfer learning remains a common strategy for handling small agricultural datasets. Varma et al.^27^ demonstrated the strength of pre-trained models such as InceptionV3 and DenseNet121 in detecting mango leaf diseases. Tian et al.^28^ used a multi-scale Dense Net combined with GAN augmentation to classify diseases in apples under various environmental conditions.

Beyond mere disease detection, deep learning is now influencing other key agricultural tasks. Azadnia et al.^29^ used CNNs to classify ripeness levels in hawthorn fruits, achieving perfect accuracy and facilitating improved post-harvest handling. Ensemble methods (VGG16, Efficient-Net, ResNet) were utilized by Novtahaning et al.^30^ for coffee leaf disease, further illustrating the power of combining multiple models to minimize misclassification and maximize generalizability. By coupling transfer learning CNNs with the bio-inspired Gravitational Search Algorithm, Al-Gaashani et al.^31^ achieved 99.12% accuracy, highlighting how meta-heuristic optimization can enhance convergence and robustness in plant disease classification. The field has also moved toward capturing temporal and real-time patterns. For example, Tiwari et al.^32^ combined graph neural networks with RNNs to detect disease progression in finger millet leaves, which enables the tracking of disease development over time. Model comparisons for tomato ripeness by Pangilinan et al.^10^ identified VGG19 as the top performer, while Bhatia et al.^33^ developed a hybrid SVM-LR approach that boosted robustness and noise resilience for powdery mildew prediction. In the citrus dataset, Asad et al.^34^ reached 94.55% accuracy using a multilayer CNN. In guava, Sharma et al.^35^ reported DenseNet169 achieving 99.62% accuracy when used with TL. Al-Gaashani et al.^36^ architecture combines attention mechanisms, multi-residual learning, and dilated spatial pyramid pooling to improve feature extraction, achieving 99.3% accuracy and establishing a new benchmark for lightweight, real-time plant disease detection.

For deployment-ready systems, Wakhare et al.^37^ created a web interface that uses CNN and SVM for pomegranate diagnosis with 98.38% accuracy. Chaturvedi et al.^11^ used EfficientNetB4 to detect tomato surface defects, while Zahra et al. ^38^ achieved high precision on apple and grape diseases using a two-stream DL model. For mango classification, Ayalew et al.^21^ used GoogLeNet and VGG16 in an ensemble framework with 99.21% accuracy, and Sankaran et al.^39^ presented CitrusDiseaseNet, combining ResNet and KELM, achieving 98.9% in citrus. The integration of language models in agricultural diagnostics is a recent but rapidly expanding area. Surveys by Zhao et al.^15^ and Singh et al.^16^ highlight Retrieval-Augmented Generation (RAG) as a powerful technique for generating disease-specific treatment recommendations, bridging the gap between detection and actionable guidance. Despite such remarkable progress, certain limitations persist. Many existing works either focus solely on binary or multi-class disease classification or do not provide a reliable estimation of disease severity, leaving a crucial gap between diagnosis and actionable intervention. Furthermore, explainability is often neglected, even though it is critical for building trust and facilitating adoption among farmers and agronomists. Additionally, only a handful of systems deliver actionable recommendations tailored to disease type and severity, and even fewer offer end-to-end deployment in a user-friendly manner.

The present study directly addresses these gaps by introducing a comprehensive, scalable solution for pomegranate disease management. The proposed system employs a DenseNet121-based CNN model fine-tuned with domain-specific augmentations, which achieves an exceptional 99.35% accuracy in classifying four major pomegranate diseases. Moving beyond traditional classification, the work presents the Healthy-Based Deviation Scoring (HBDS) method, which uniquely combines Grad-CAM ++ and Mahalanobis distance for explainable, quantitative severity estimation, outperforming pixel-threshold approaches and aligning closely with expert annotation. Finally, this diagnostic pipeline is augmented with a domain-specific, RAG-powered recommendation module that delivers severity-aware, scientifically validated treatment plans via an accessible web application.

In summary, by uniting high-accuracy classification, interpretable severity scoring, and real-time, actionable recommendations in a single deployable framework, this work sets a new standard for precision agriculture solutions, bridging the longstanding gap between disease detection, severity assessment, and practical field support. Table 1 lists the previous studies related to disease detection in various plants.

**Table 1:** Overview of research papers reviewed in study.

References	Technique used	Dataset	Outcome of the work
Ishak Pacal et al.^7^	CNN and Vision Transformer (ViT)	PlantVillage dataset (4062 images), Grapevine dataset (500 images)	Achieved perfect accuracy in grape leaf disease and variety classification using CNN and ViT models
Ayush Gupta et al.^8^	Extraction of features using CNN, Random Forest for classification	Dataset consists 4,579 pomegranate fruit images with six health categories	The hybrid model achieved 91.59% accuracy in classifying pomegranate diseases
John Raphael Pangilinan et al.^10^	InceptionV3, ResNet50, VGG19 (CNN Models)	Tomato ripeness dataset: 1260 images, 6 ripeness stages	VGG19 reached 95% accuracy, outperforming ResNet50 at 93.33% and InceptionV3 at 91.67%
Akshat Chaturvedi et al.^11^	VGG19, ResNet50, DenseNet201, EfficientNetB4, InceptionV3 (CNN-based models)	Healthy and defective tomatoes of 43,843 images	EfficientNetB4 outperformed other models with 97.97% accuracy in defect detection
Aleka Melese Ayalewet al.^21^	Ensemble CNN (GoogLeNet, VGG16)	3195 mango images (Amhara region)	Achieved 99.21% testing accuracy for mango disease classification
Yohannes Agegnehu Bezabh et al.^22^	A hybrid approach using GLCM, filtering, and multiple classifiers for classification	1247 chickpea leaf images from Gondar, Ethiopia, expanded to 3330	Achieved 95.49% accuracy with SVM classifier using GLCM-Color Histogram features
Pradeep B. Maneet al.^23^	CNN	Collected dataset of infected and healthy pomegranate images	Achieved 92% accuracy in detection, 89% in classification, enabling deployment
Jiwen Yang et al.^26^	CNNs for deep feature extraction, integrated with SVM, Random Forest, and MLP	4669 cherry images in 5 categories from Xi’an, China orchard	MobileNet_v2 with SVM achieved 98.3% accuracy for cherry disease detection
Teena Varma et al.^27^	Transfer learning using InceptionV3, VGG19, ResNet152V2, DenseNet121, InceptionResNetV2, MobileNetV2, Xception	Mango leaf dataset (4000 images)	InceptionV3 performed best with 99.87% accuracy
Yunong Tian et al.^28^	Multi-scale Dense Classification Network, DenseNet, Inception-V4, Inception-ResNet-V2, Cycle-GAN	Healthy and diseased apple leaves and fruits, with Cycle-GAN images of 11 categories	Inception-ResNet-V2, which has a multi-scale dense layer, achieved 94.74% accuracy in diagnosing apple diseases
Damar Novtahaning et al.^30^	Ensemble model (VGG-16, EfficientNet-B0, ResNet-152)	Coffee Leaf Disease Dataset: 1300 images (Cercospora, Phoma, Miner, Rust, Healthy)	The ensemble method achieved 97.31% accuracy in coffee disease detection
Anshul Bhatia et al.^33^	A hybrid SVM-LR classifier used ANR for noise reduction, boosting accuracy	TPMD dataset balanced using Random Oversampling	Hybrid SVM-LR achieved 92.37% accuracy, surpassing SVM and LR models
Asad et al.^34^	Multilayer CNN	Citrus and PlantVillage datasets (2293 images)	Achieved 94.55% accuracy in detecting citrus fruit and leaf diseases
Sanjeev Sharma et al.^35^	CNN with Transfer Learning (DenseNet, ResNet, VGG, MobileNet, Inception)	Dataset of 681 guava fruit and leaf images across 6 classes	DenseNet169 outperformed previous models by achieving the highest test accuracy of 99.62% for guava disease detection
Prashant Wakhare et al.^37^	CNN and SVM combination	Kaggle dataset of pomegranate images (3976 total images, including healthy and diseased)	Achieved 98.38% accuracy in identifying pomegranate diseases using a web-based tool
Unber Zahra et al.^38^	Two-stream deep learning with Inception-ResNet-V2 and tree growth optimization for feature selection	Plant Village dataset (apple and grape diseases)	Achieved 99.9% accuracy for apple and 99.4% for grape disease detection
Shanmugapriya Sankaran et al.^39^	CitrusDiseaseNet: Integrated CNN ResNet and KELM	Citrus fruit and leaf dataset with 21,807 images for training/testing	Achieved 98.9% accuracy, outperforming existing citrus disease detection methods
Biniyam Mulugeta Abuhayi et al.^40^	Hybrid approach using ResNet and LeNet CNN architectures	Pumpkin leaves with 2646 augmented images and fruits from Ethiopia	The hybrid CNN model achieved 99.78% training, 98.18% validation, and 97.21% testing accuracy

## Methodology

**Fig. 1:**
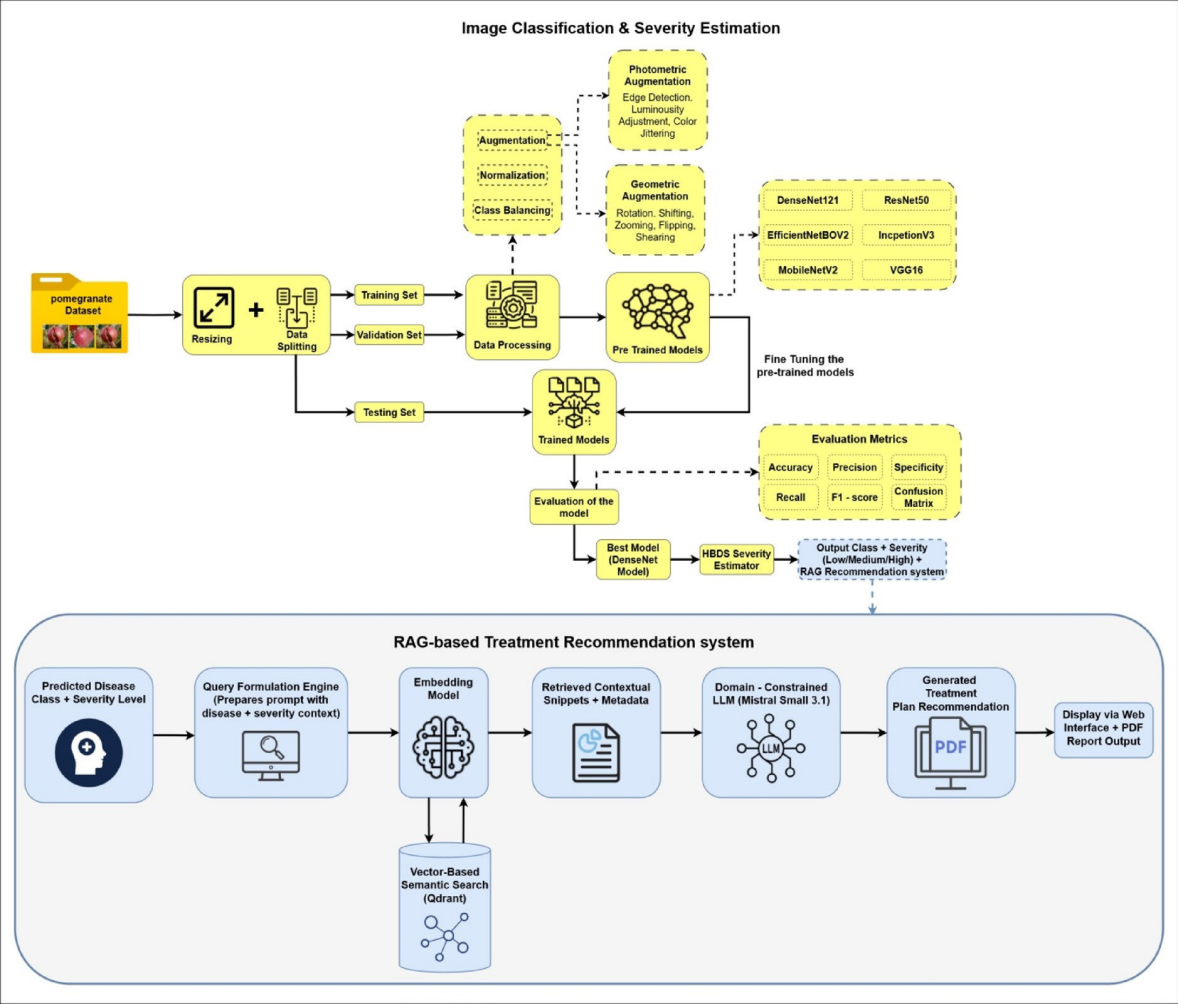
Architecture of proposed work.

This section elaborates on the comprehensive methodology designed to detect and assess the Pomegranate Fruit Diseases Dataset using deep learning. The workflow, illustrated in Fig. 1, involves preprocessing a labelled fruit image dataset, training CNN-based classifiers via transfer learning, estimating severity using the HBDS framework, and integrating a Retrieval-Augmented Generation (RAG) based system to provide treatment recommendations. Each phase was optimized to enhance performance, interpretability, and applicability in real-world agricultural environments.

### Dataset and preprocessing

This study utilizes the publicly available Pomegranate Fruit Diseases Dataset for Deep Learning Models, which comprises 5099 high-resolution images of pomegranate fruits annotated by domain experts^5^. The dataset was collected during the July–October 2023 harvest season from farms in Karnataka, India, using a Redmi 9 smartphone camera (3120 × 3120 pixels) under diverse natural lighting and environmental conditions to ensure ecological robustness. Each image belongs to one of five categories: Healthy, Alternaria Fruit Spot, Bacterial Blight, Anthracnose, or Cercospora Fruit Spot. Expert annotation was conducted by the Indian Institute of Horticultural Research (IIHR) in collaboration with the Indian Council of Agricultural Research (ICAR), ensuring high reliability. The Pomegranate fruit diseases dataset is hosted on Mendeley Data and has been formally published in *Data Brief*^5^, making it freely accessible under a Creative Commons Attribution 4.0 License (CC BY 4.0).

**Fig. 2:**
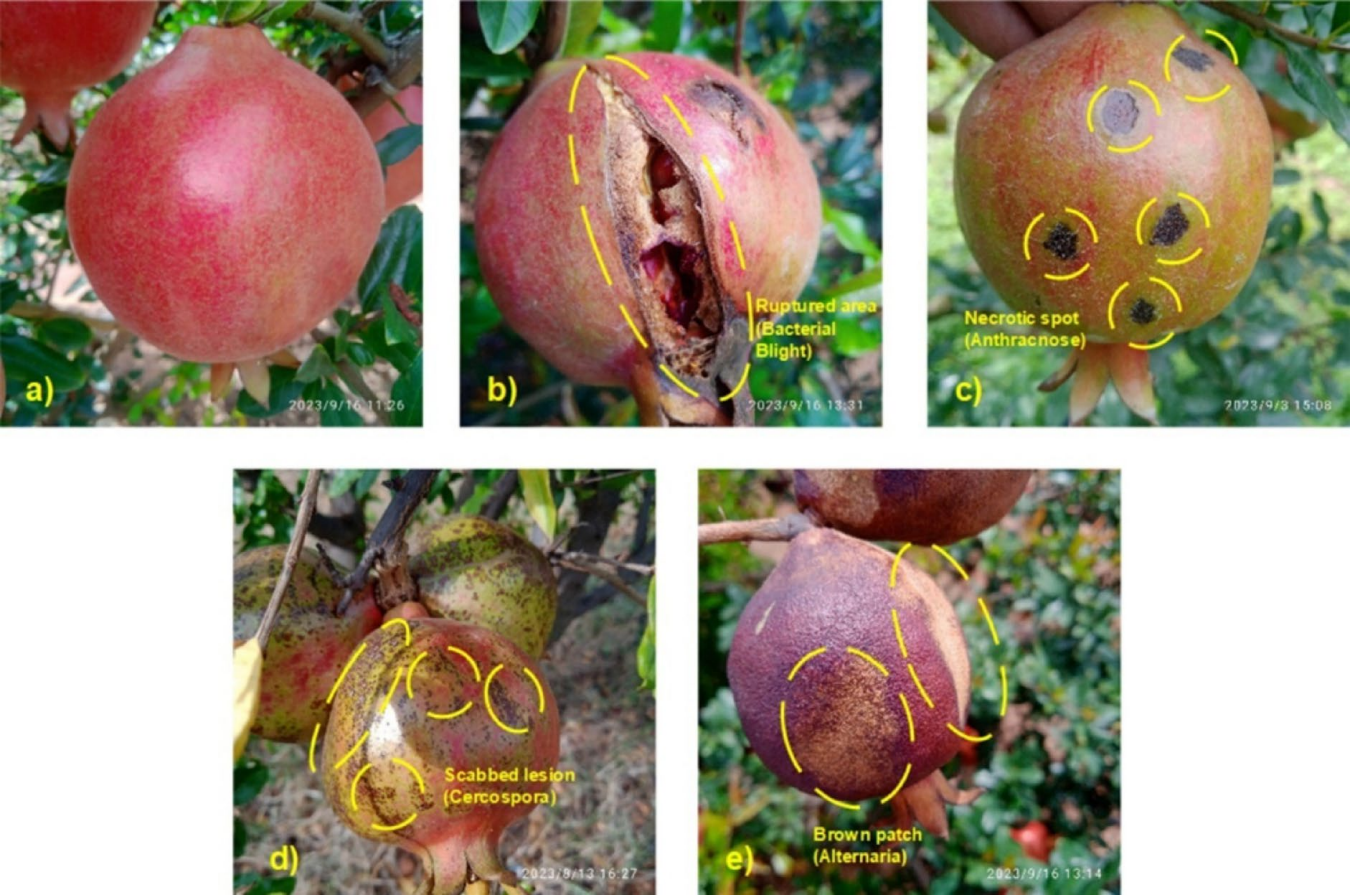
Sample images of pomegranate fruit diseases dataset for deep learning model [https:// data.mendeley.com/datasets/b6s2rkpmvh/1], used under the Creative Commons Attribution 4.0 License (CC BY 4.0). (**a**) Healthy, (**b**) Bacterial Blight, (**c**) Anthracnose (**d**) Cercospora (**e**) Alternaria.

From a pathological perspective, the selected classes represent distinct visual and morphological manifestations of both fungal and bacterial infections. Healthy fruits exhibit smooth, lustrous skins ranging from greenish yellow to red brown (Fig. 2a). Bacterial Blight (Xanthomonas axonopodis pv. punicae) appears as sunken, dark lesions often leading to severe outbreaks (Fig. 2b), while Anthracnose (Colletotrichum spp.) manifests as concentric ring-shaped spots under humid conditions (Fig. 2c). Cercospora punicae leads to cracked brown lesions (Fig. 2d), and Alternaria alternate causes black, velvety-textured patches (Fig. 2e). As documented in a prior study^5^, these infections, especially Bacterial Blight and Anthracnose, significantly impair fruit quality and market value if not diagnosed early. To prepare the dataset for model training, a structured preprocessing pipeline was employed, comprising five critical stages: dataset partitioning, image resizing, augmentation, class imbalance correction, and normalization. First, the dataset was randomly shuffled and partitioned into 70% training, 15% validation, and 15% testing splits as in Table 2. This distribution is widely validated in precision agriculture literature^41^ and was adopted here to facilitate reliable generalization and performance benchmarking.

**Table 2:** Dataset split.

Classes	Total no. of images	No. of training images	No. of validation images	No. of testing images
Alternaria	886	620	132	134
Anthracnose	1166	816	174	176
Bacterial blight	966	676	144	146
Cercospora	631	441	94	96
Healthy	1450	1014	217	219

Subsequently, all images were resized to 224 × 224 pixels to align with the input dimensionality requirements of deep learning architectures such as DenseNet121, ResNet50, and MobileNetV2. Resizing not only harmonizes model compatibility but also accelerates convergence by reducing computational load, especially when leveraging transfer learning from ImageNet pre-trained weights^42^.

Data augmentation artificially increases the diversity of the training data by applying transformations to the original images, thereby helping to prevent overfitting and improve generalization. Let $x$ be an original image, and $T$ be a transformation function (e.g., rotation, flipping, scaling). The augmented image $x{\prime}$ is obtained as:

1 $${x}{\prime}=T\left(x\right)$$

Common transformations include rotation, expressed as ${T}_{rot}(x,\theta )$ which rotates the image by an angle $\theta$, horizontal flipping, expressed as:

2 $${T}_{flip}\left(x\right)= {x}_{flipped}$$

and scaling, expressed as ${T}_{scale}(x,s)$ which scales the image by a factor $s$.

To further enhance model robustness, a two-tier augmentation strategy was applied. Standard geometric augmentations included random rotation (± 20°), zooming (up to 20%), horizontal/vertical flipping, width and height shifting (20%), and shearing. These transformations artificially increased dataset diversity and helped simulate real-world image capture variations such as hand movement, angle deviation, or fruit orientation. Such strategies have shown efficacy in various horticultural CNN pipelines^35^. Complementing this, custom photometric augmentations were integrated to emphasize disease-specific visual features. Canny edge detection was employed to highlight lesion boundaries typical of Anthracnose and Cercospora^43^. The resulting edge map was fused with the original image to enhance lesion contrast. Additionally, brightness and contrast modulation (0.8–1.2 scaling) simulated different sunlight conditions, while color jittering altered hue, saturation, and brightness values to improve the model’s adaptability to diverse orchard environments. These augmentations align with the domain shifts encountered in field deployments and draw upon similar approaches reported in citrus-based detection systems^34^.

Class imbalance, a standard limitation in plant disease datasets, was addressed via weighted loss functions. To handle class imbalance, class weights were computed and applied, ensuring minority classes contributed proportionally to the loss function. The weight for each class $i$ was calculated as:

3 $${w}_{i}\frac{N}{{n}_{i}}$$

where ${w}_{i}$ represents the class weight for class, $i,N$ is the total number of samples, and ${n}_{i}$ is the number of samples in class $i$. The relative frequency of underrepresented classes, particularly Alternaria and Cercospora, was used to adjust the loss function during training, thereby reducing model bias toward dominant categories. Previous studies have demonstrated the significance of such strategies in both classical^44,45^ and deep learning-based pomegranate disease classification tasks. Lastly, all image pixel values were normalized to the [0, 1] range by dividing by 255.

Normalization scales input data to a standard range or distribution, thereby improving convergence during training. Given an input image tensor $X$ with pixel values, normalization is typically defined as:

4 $${X}_{norm}=\frac{X- \mu }{\sigma }$$

where $\mu$ and $\sigma$ denote the mean and standard deviation of the dataset or batch, respectively. In practice, this process often includes rescaling, where pixel intensities are mapped to [0, 1] range by dividing by 255 for 8-bit images:

5 $${X}_{rescaled}=\frac{X}{255}$$

Followed by mean subtraction and standardization as expressed in the normalization formula above. Maintaining numerical stability during backpropagation, improving model convergence, and avoiding scale-induced bias during weight updates depend on this phase. Using CNNs in batch-based training depends primarily on normalizing to ensure that input tensors remain within a stable range^46^. These preprocessing steps, taken together, transformed the raw image information into a robust input pipeline that maximized the performance of the classification and severity estimation models discussed in the following sections.

### CNN-Based disease classification using transfer learning

Transfer learning has evolved into a crucial method in agricultural image categorization, particularly in situations with little labeled data. It uses generalized feature representations learnt across several picture domains by reusing weights from models trained on large-scale datasets like ImageNet. This work applied transfer learning to provide strong and scalable pomegranate disease categorization using five Healthy categories: Alternaria, Anthracnose, Bacterial Blight, and Cercospora. Six popular CNN architectures were fine-tuned for the task: DenseNet121, EfficientNetB0V2, MobileNetV2, ResNet50, VGG16, and InceptionV3. All models were trained on the preprocessed pomegranate dataset, starting with ImageNet weights and modified via transfer learning by unfreezing the top layers. The chosen models were picked depending on their architectural efficiency^47^ and proven effectiveness in tasks related to agricultural disease identification^48^.

DenseNet121 was chosen for its dense connectivity mechanism that enhances gradient flow and feature reuse. With only 7.6 M trainable parameters, it offered a favourable balance between depth and performance, especially on class-imbalanced datasets. EfficientNetB0V2 incorporated compound scaling and attention mechanisms, yielding high accuracy with minimal computational cost. MobileNetV2, designed for mobile and edge deployment, provided fast inference with depthwise separable convolutions, making it suitable for real-time agricultural diagnostics^45^. ResNet50, known for its skip connections, facilitated deeper representation learning and alleviated vanishing gradient issues in deeper architectures. It was included to evaluate how residual learning benefits fruit disease classification. VGG16, though older, remains a strong baseline due to its uniform layer structure and ease of tuning. Finally, InceptionV3 introduced multi-scale convolutional operations, enabling the network to capture fine-grained disease patterns at varying receptive fields. These models, when fine-tuned with domain-specific augmentation and balanced training practices, will provide a strong baseline for evaluating CNN performance in agricultural scenarios. Comparative analysis of these architectures helps identify the most efficient configuration for deployment in real-world pomegranate farming applications.

### Hyperparameter configuration and model comparison

All CNN models were trained under consistent hyperparameter settings to ensure fair performance comparison. The Adam optimizer was used with a learning rate of 1e^−5^, a batch size of 8, and a fixed epoch count of 60. Categorical cross-entropy served as the loss function for this multi-class classification task, and early stopping (patience: 5) with ReduceLROnPlateau (factor: 0.5, patience: 5) was employed to prevent overfitting and ensure smooth convergence. These decisions were informed by prior benchmarks on similar image classification tasks. Regularization was enforced using a dropout rate of 0.3 in the dense layers and L2 regularization (0.001) on weights. These strategies reduced overfitting and enhanced generalization, especially in the presence of complex disease textures. Additionally, class weights were calculated and provided during training to mitigate bias toward overrepresented classes, addressing the skewed distribution of disease classes^44,45^.

Model performance was also evaluated in terms of architectural efficiency. Metrics such as the number of trainable and non-trainable parameters, model size (MB), and total training time (seconds) were recorded. DenseNet121 and EfficientNetB0V2 offered favourable trade-offs between accuracy and size, while MobileNetV2 showed the best efficiency for edge deployment. ResNet50 and InceptionV3, despite longer training times, achieved stable performance, whereas VGG16 required moderate training time but demonstrated effective generalization. These results, detailed in section “Model evaluation and result discussions”, underscore how different architectural trade-offs influence model selection for real-world deployment, with DenseNet121 emerging as a strong candidate in terms of robustness, speed, and accuracy. Table 3 gives the hyperparameter specification for the models used.

**Table 3:** Specification of hyperparameters for the models.

Hyperparameter	Value
Batch size	8
Learning rate	1e^−5^
Epochs	60
Optimizer	Adam
Dropout rate	0.3
Weight decay (Regularizer)	L2 regularization, 0.001
Class weights	Provided to handle the imbalance
Early stopping	Patience: 5
Learning rate reduction	ReduceLROnPlateau (factor: 0.5, patience: 5)
Loss function	Categorical cross-entropy

### Severity estimation using HBDS

**Fig. 3:**
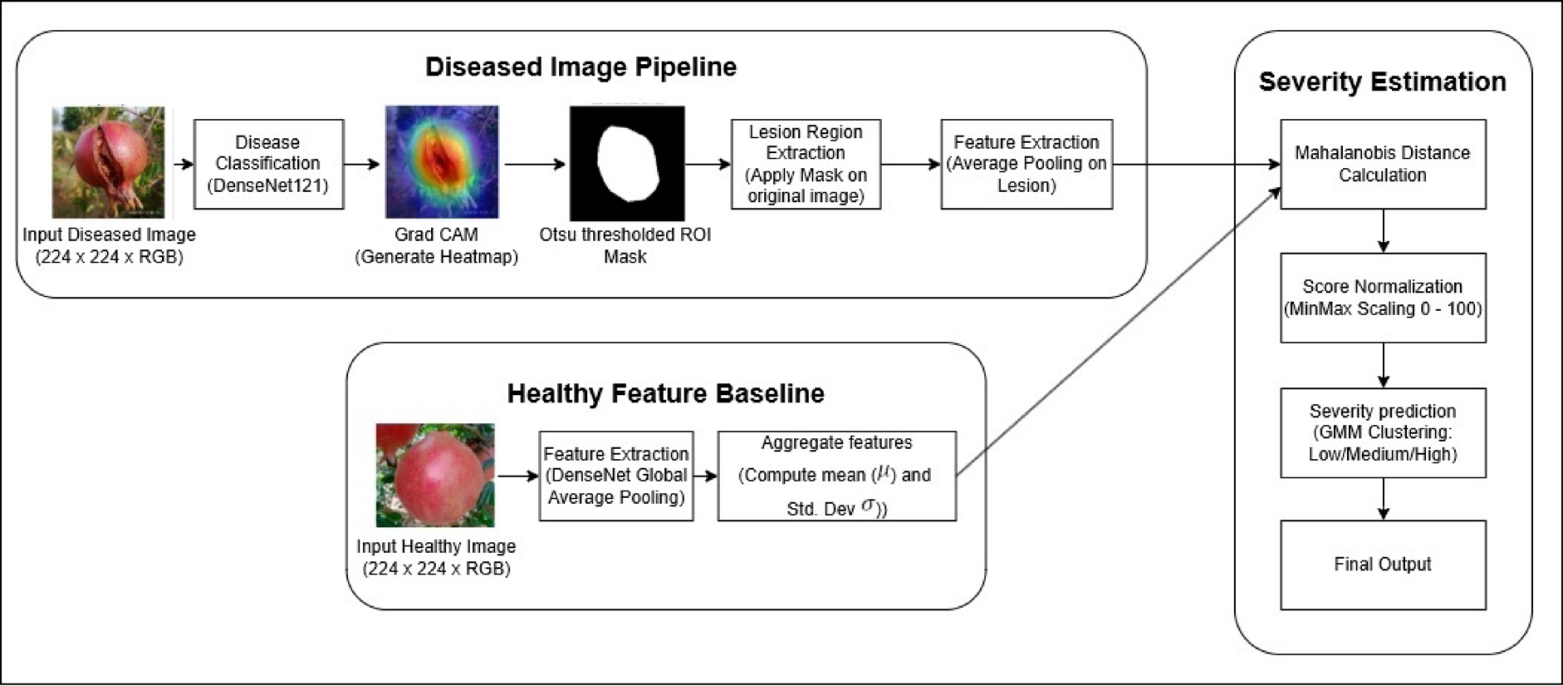
HBDS pipeline for pomegranate disease severity estimation using grad-CAM ++ and mahalanobis distance created by the authors.

While classification enables the identification of disease type in pomegranate fruits, real-world agricultural decision-making often hinges on understanding the severity of infection. To meet this need, a post-classification framework named Healthy-Based Deviation Scoring (HBDS) is proposed (Fig. 3). Inspired by visual inspection methods used in manual assessment, HBDS introduces a statistically grounded and explainable strategy to quantify the progression of infection from healthy baselines and ultimately categorize each sample into one of three interpretable severity levels: Low, Medium, or High. The HBDS pipeline is initialized after disease classification by DenseNet121 (which emerged as the best model), which is repurposed to act as a feature extractor. Deep embeddings are extracted from the avg_pool layer, yielding 1024-dimensional vectors that encapsulate mid-level and high-level semantic features associated with both healthy and diseased fruit images. For this study, a reference embedding distribution was created using 1450 clean samples from the Healthy class, serving as a statistical baseline.

**Fig. 4:**
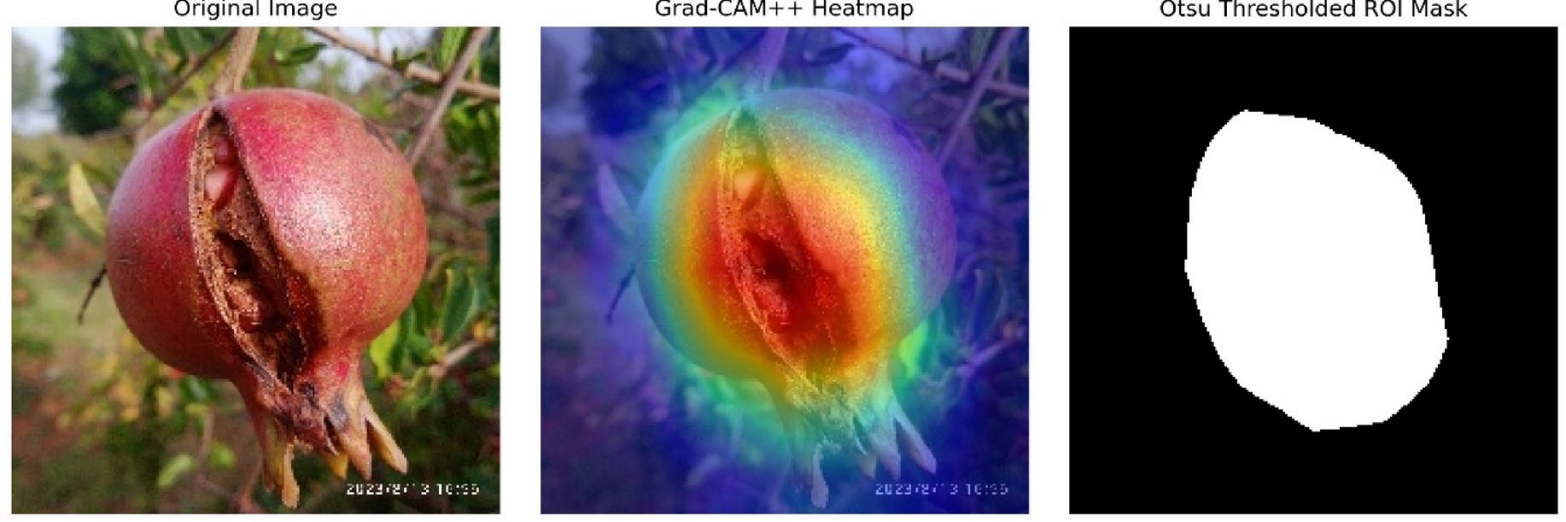
Grad-CAM++-based lesion localization and Otsu-thresholded ROI visualization generated by the authors as part of the explainable deep learning module for severity estimation.

From this reference set, a feature-wise distribution was constructed by calculating the mean (μ_i_) and standard deviation (σ_i_) across all dimensions. These healthy distributions form the foundation of our deviation scoring mechanism, in line with the statistical anomaly detection principle used in plant pathology studies^49^. To isolate diseased regions within each test image, Grad-CAM ++ as applied to generate class-discriminative saliency maps. Compared to Grad-CAM, the Grad-CAM ++ method produces more localized and fine-grained activations, making it especially useful in highlighting lesion-specific regions in fruit imagery. These heatmaps were further binarized using Otsu’s thresholding (Fig. 4), a robust unsupervised method for identifying bimodal intensity thresholds, as seen in complex agricultural images^41^.

The segmented lesion region is cropped and re-passed through the feature extractor to obtain lesion-specific embeddings. To quantify how much a given lesion diverges from healthy standards, the Mahalanobis distance is calculated between the lesion embeddings and the healthy feature distribution. Let ${f}_{lesion}\in {\mathbb{R}}^{d}$ represent the feature embedding of a lesion ROI obtained from the DenseNet 121 network, and let ${\mu }_{\text{H}}$ and ${\sum }_{\text{H}}$ denote the mean vector and covariance matrix derived from the healthy feature bank. The Mahalanobis Distance ${D}_{\text{M}}$ of the lesion embedding from the healthy distribution is computed as:

6 $${D}_{\text{M}}\left({f}_{lesion}\right)= \sqrt{{\left({f}_{lesion}-\mu {\rm H} \right)}^{\text{T}}{\Sigma }_{\rm H}^{-1}\left({f}_{lesion}-\mu {\rm H} \right) }$$

This distance metric is favoured over simple Euclidean alternatives due to its ability to incorporate inter-dimensional correlation, which has been proven beneficial for medical and agricultural imaging contexts^50^. To unify the score range across samples and classes, the computed Mahalanobis distances are normalized via Min–Max scaling to a 0–100 range. This transformation standardizes the interpretation of deviation scores and ensures compatibility with probabilistic models. The normalized values are clustered using Gaussian Mixture Models (GMM) into three severity levels. GMM was chosen for its capacity to model soft probabilistic boundaries between clusters, which better reflect real-world disease progression compared to hard classifiers like K-Means^32^.

**Fig. 5:**
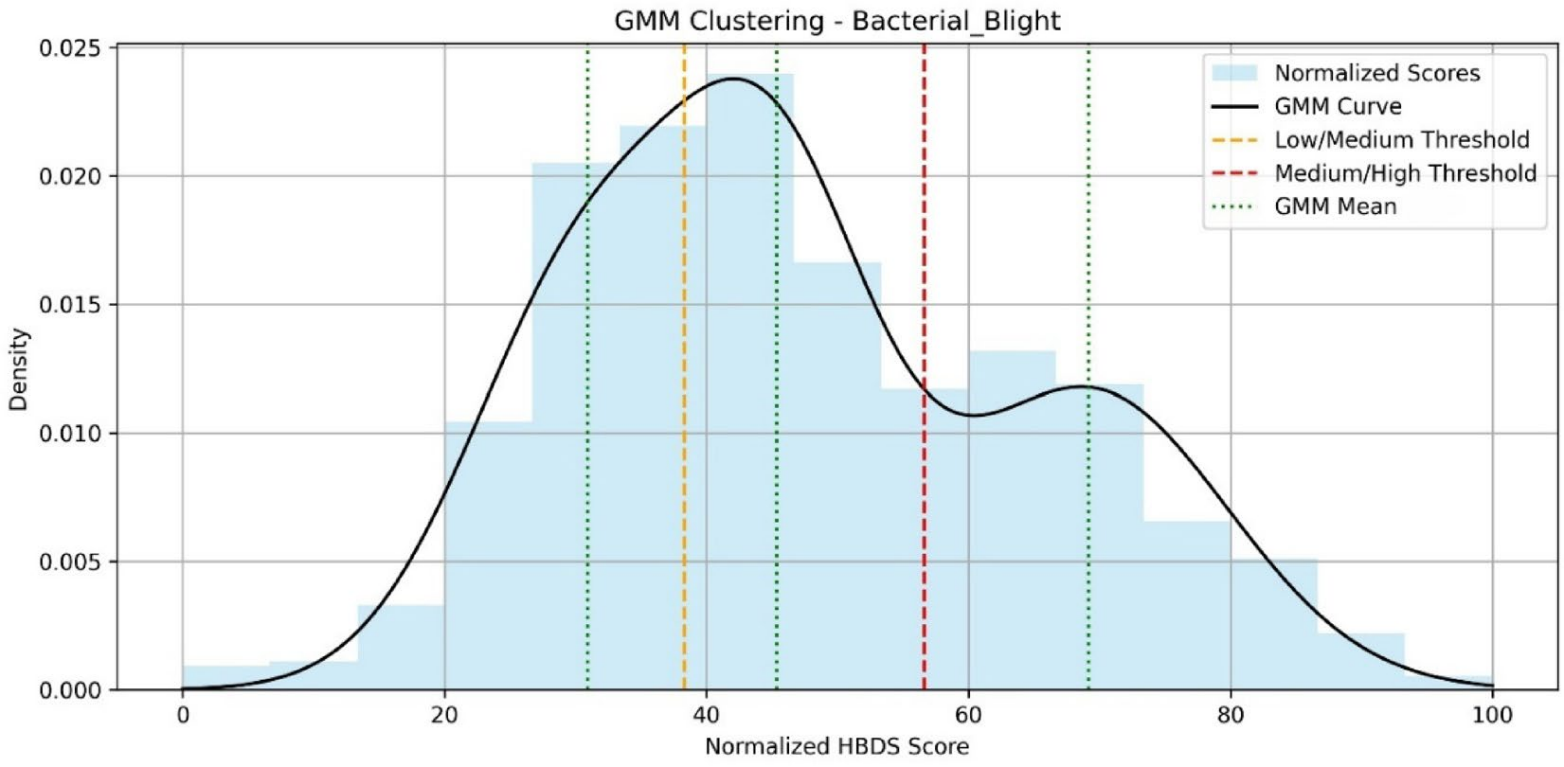
GMM-based severity clustering for bacterial blight using normalized HBDS scores.

This integrated HBDS mechanism effectively shifts disease classification from discrete labelling to a more nuanced understanding of how severe a particular infection is. It offers a practical advantage in the field by enabling early-stage interventions for low-risk cases and prioritizing treatment for high-severity infections. In Fig. 5, the bacterial disease impact was classified into three levels: Low (0–38%), Medium (38–58%), and High (58–100%) to systematically evaluate and quantify its extent, guided by expert assessment. Like this, each disease has separate Low, Medium, and High scales done by GMM clustering.

### Code availability

The source code implementing the pomegranate disease diagnosis framework (deep-learning classifier, severity estimation module, and RAG-based treatment recommender), together with trained weights, inference scripts, and environment files, has been deposited in an open repository and is available at 10.5281/zenodo.17318797. The archived release corresponds to the exact version used in this study (tag: v1.0; commit: [short hash]) and includes a sample input and reproducibility instructions. The pomegranate image dataset is externally hosted under CC BY 4.0 (see Data Availability) and is not bundled with the code.

## Model evaluation and result discussions

### Performance evaluation metrics

All model training and evaluation were conducted using an NVIDIA RTX 3050 GPU with 16 GB VRAM. The implementation was carried out in Python using TensorFlow and Keras libraries. Model checkpoints and logs were stored using TensorBoard for visualization of training dynamics such as loss curves and accuracy trends. Random seeds were fixed across Numpy, TensorFlow, and Python environments to ensure reproducibility of results. The model trained should classify and detect the pomegranate disease correctly and accurately. For this, some performance metrics were employed to assess their efficiency in identifying the diseases. These metrics include precision, accuracy, F1-score, recall, and specificity. With the help of these metrics, the model is evaluated on its ability to precisely classify images of healthy and diseased pomegranates, including those affected by Alternaria, Anthracnose, Bacterial Blight, and Cercospora. The following subsections provide details of each metric concerning the pomegranate disease classification.

*Accuracy* It is the measure that shows how the model has correctly identified pomegranate disease cases (both true positives and true negatives) across all samples shown in Eq. (7). In general, this statistical measure shows how well the model can tell the difference between healthy and sick pomegranates.

7 $$\text{Accuracy}= \frac{\text{ True Positives}\left(\text{TP}\right)+\text{True Negatives}\left(\text{TN}\right)}{\text{Total Number of Instances}}$$

*Precision* As shown in Eq. (8), it is the metric that depicts how many of the images of diseased pomegranates were correctly identified as it has been labeled. This is particularly important to minimize the misclassification of healthy pomegranates as diseased, which in turn reduces unnecessary interventions.

8 $$\text{Precision}=\frac{\text{ TP}}{\text{TP}+\text{FP}}$$

*Recall (Sensitivity)* It is a measure that calculates how well the model can identify true positive cases among all the real sick pomegranates. This ensures that the model correctly identifies all sick pomegranates, which is essential for managing diseases quickly and effectively.

9 $$\text{Recall}=\frac{\text{ TP}}{\text{TP}+\text{FN}}$$

The macro-averaging technique is used in this work to calculate recall. At first, it is calculated independently for each class, and then an average of the results is calculated. This approach ensures equal weight for all pomegranate disease categories, preventing performance from being dominated by majority classes and effectively addressing the challenges of class imbalance.

*F1-score* It is the score that is calculated as the harmonic mean of recall and precision. It provides a balanced metric that evaluates the trade-off between detecting diseased pomegranates accurately and minimizing false positives, as shown in Eq. (10).

10 $$\text{F1-}\text{score}=2\times \frac{\text{ Precision}\times \text{Recall}}{\text{Precision}+\text{Recall}}$$

*Specificity* This evaluates the model’s capacity to identify healthy pomegranates among all actual healthy cases correctly. High specificity is very crucial for avoiding false alarms in healthy crops. It optimizes the efficiency of disease interventions.

11 $$\text{Specificity}=\frac{\text{ TN}}{\text{TN}+\text{FP}}$$

Like recall, specificity is calculated for each class using the macro-averaging approach. This guarantees that every class is handled identically by offering a fair assessment of the model’s capacity to handle several classes, irrespective of their respective shares in the dataset.

### Model classification performance

**Fig. 6:**
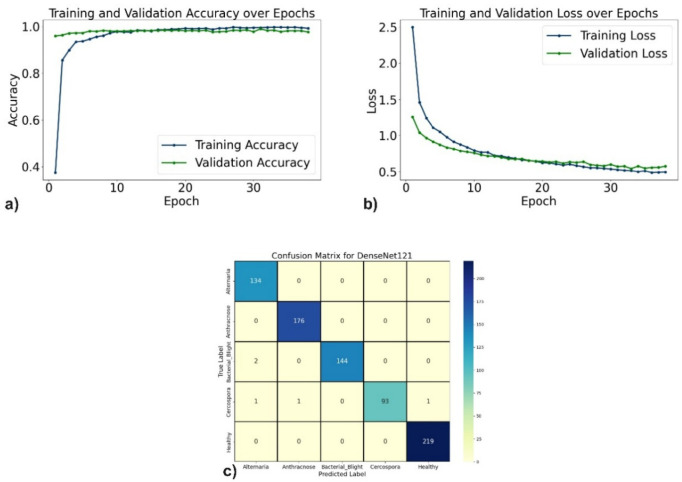
(**a**) Accuracy graphs over 40 epochs on training data (**b**) Loss graphs over 40 epochs on training data (**c**) Confusion matrix for Densenet121.

**Fig. 7:**
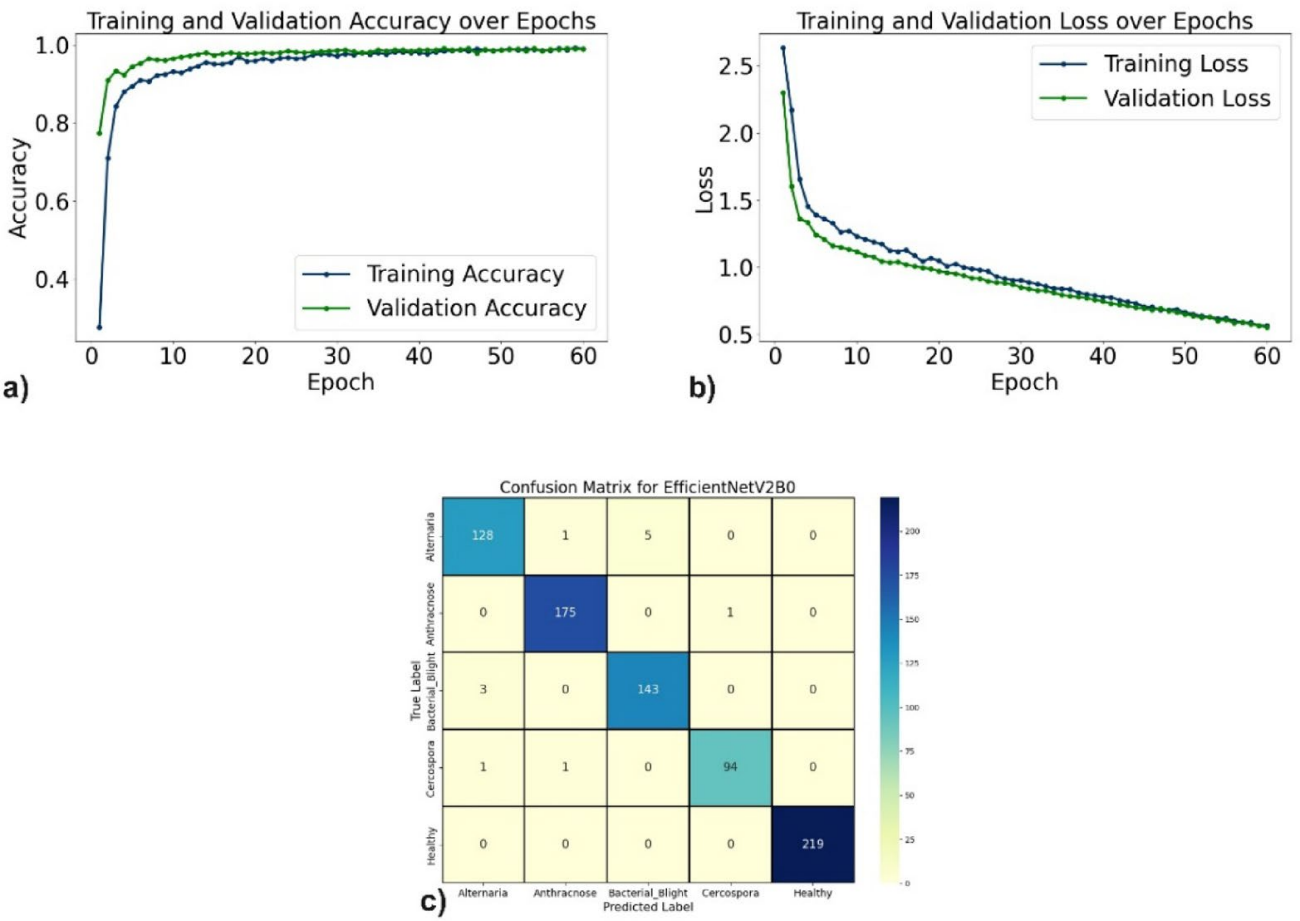
(**a**) Accuracy graphs over 60 epochs on training data (**b**) Loss graphs over 60 epochs on training data (**c**) Confusion matrix for EfficientNetV2B0.

**Fig. 8:**
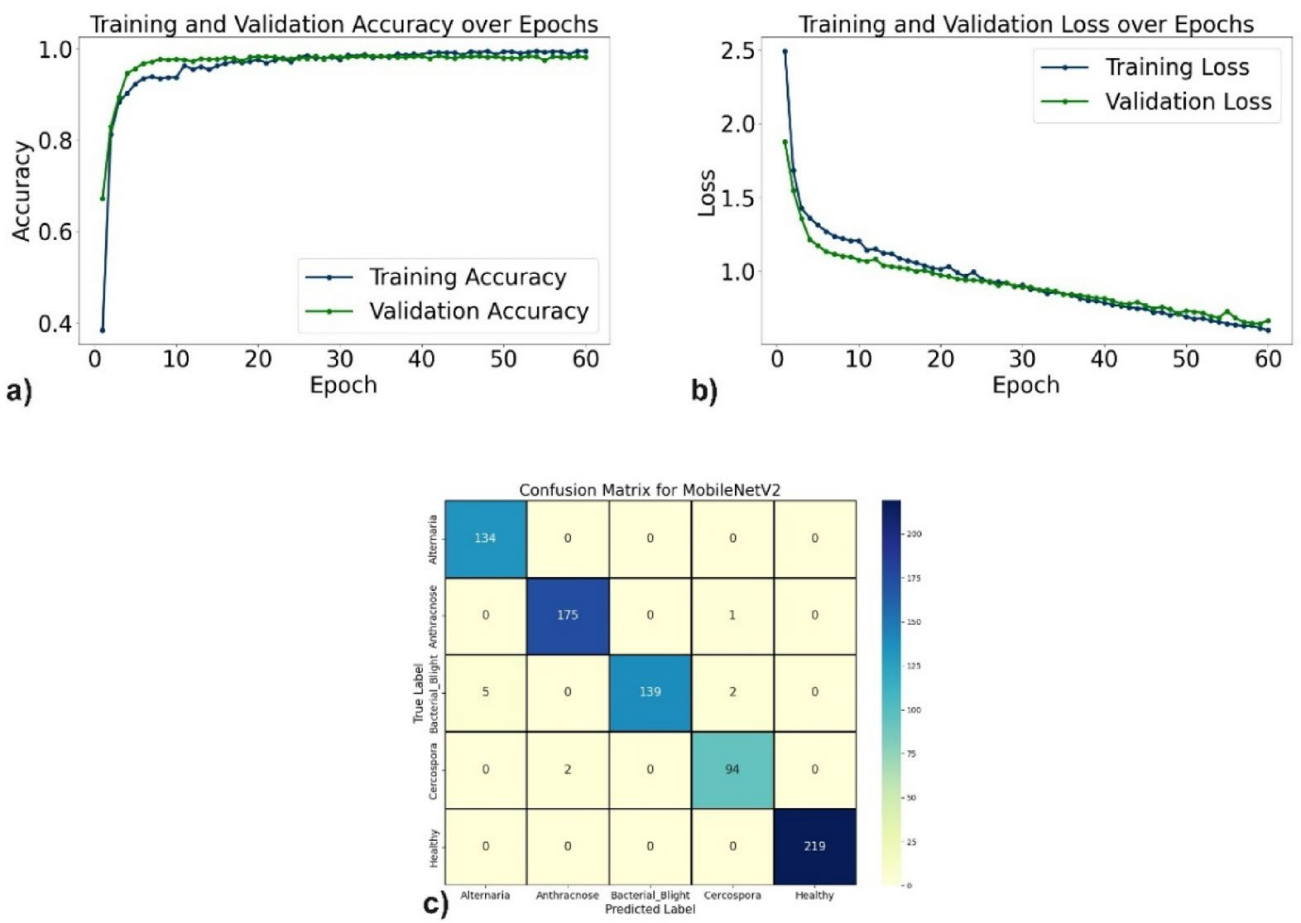
(**a**) Accuracy graphs over 60 epochs on training data (**b**) Loss graphs over 60 epochs on training data (**c**) Confusion matrix for MobileNetV2.

**Fig. 9:**
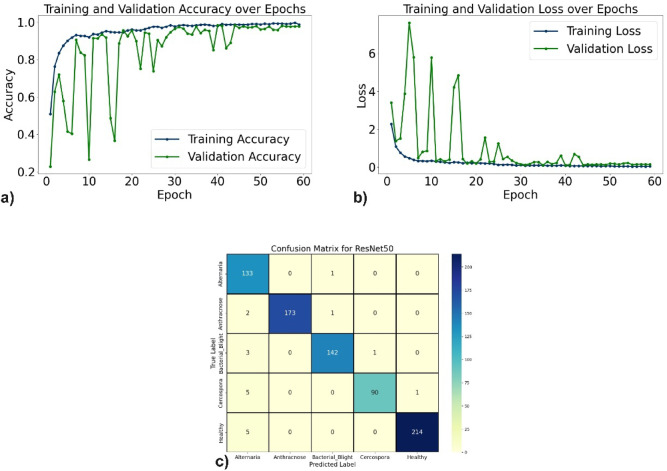
(**a**) Accuracy graphs over 60 epochs on training data (**b**) Loss graphs over 60 epochs on training data (**c**) Confusion matrix for ResNet50.

**Fig. 10:**
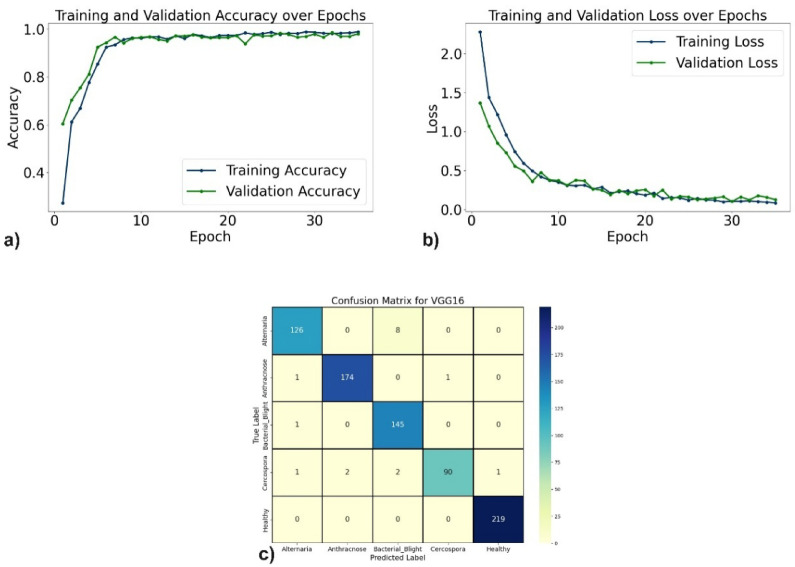
(**a**) Accuracy graphs over 35 epochs on the training data (**b**) Loss graphs over 35 epochs on the training data (**c**) Confusion matrix for VGG16.

**Fig. 11:**
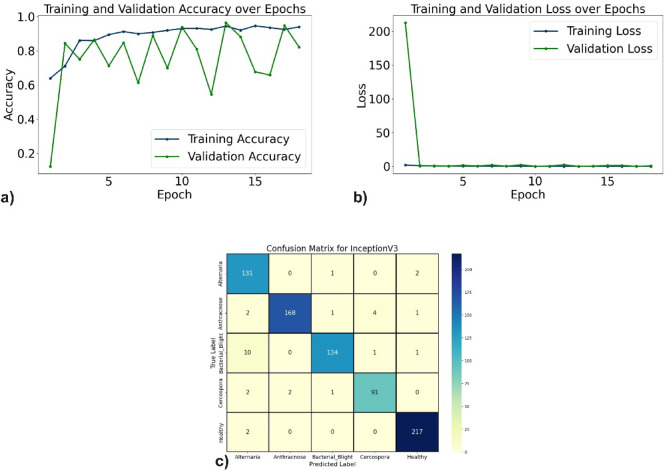
(**a**) Accuracy graphs over 20 epochs on training data (**b**) Loss graphs over 20 epochs on training data (**c**) Confusion matrix for InceptionV3.

The evaluation of the proposed DL models for pomegranate disease detection highlights their effectiveness across multiple performance metrics, including accuracy, precision, recall, F1-score, specificity, and loss. DenseNet121 emerged as the top-performing model, achieving 99.35% accuracy, 99.35% precision, and 99.10% recall, with minimal misclassification. The learning curves (Fig. 6a, b) demonstrate consistent improvement, and the confusion matrix (Fig. 6c) shows near-perfect classification for diseases such as Anthracnose and Bacterial Blight. EfficientNetV2B0 followed with 98.44% accuracy, although minor misclassifications occurred, particularly between Alternaria and Bacterial Blight. The learning curves, accuracy and loss curves are presented in Fig. 7a and b, and the confusion matrix is shown in Fig. 7c. MobileNetV2 also demonstrated strong performance, with an accuracy of 98.70%, precision of 98.83%, and recall of 98.51%, supported by stable learning curves (Fig. 8a, b) and confusion matrix (Fig. 8c). ResNet50, VGG16, and Inceptionv3 performed slightly lower but remained competitive. ResNet50 achieved 97.54% accuracy, though learning curve fluctuations indicated slight overfitting (Fig. 9a, b), as shown in the confusion matrix presented in Fig. 9c. VGG16 achieved 97.80% accuracy with steady learning (Fig. 10a, b), as shown in the confusion matrix in Fig. 10c. In contrast, Inceptionv3 had the lowest performance, with 96.11% accuracy, struggling with diseases like Cercospora (Fig. 11a, b), as displayed in the confusion matrix in Fig. 11c.

Table 4 presents a performance evaluation of the different models used. DenseNet121 stands out as the top performer, surpassing EfficientNetV2B0 and MobileNetV2, with its architecture offering high classification accuracy, precision, recall, and F1-score. Its capability to minimize misclassification and handle complex disease detection highlights its suitability for real-world applications requiring rapid and accurate results.

**Table 4:** Specification of hyperparameters for the models.

Models	Accuracy (%)	Precision (%)	Recall (Sensitivity) (%)	F1-score (%)	Specificity (%)	Loss (%)
DenseNet121	99.35	99.35	99.10	99.35	99.84	0.5183
EfficientNetV2B0	98.44	98.44	98.16	98.44	99.69	0.5711
MobileNetV2	98.70	98.83	98.51	98.70	99.69	0.6318
ResNet50	97.54	97.53	97.26	97.57	99.40	0.1113
VGG16	97.80	98.04	97.26	97.57	99.40	0.1159
InceptionV3	96.11	96.48	97.26	97.57	99.40	0.1798

Compared to earlier studies, such as the CNN-SVM hybrid model^35^ with 98.38% accuracy and traditional CNN models^14^ with 92% accuracy, DenseNet121 offers a significant improvement. Its success can be attributed to transfer learning and custom data augmentation techniques such as edge detection and luminosity adjustments, which enhanced model robustness in varying conditions, including different lighting and disease presentations.

The proposed best-performing model achieved an accuracy of 99.35% on the Pomegranate fruit diseases dataset, outperforming several baseline approaches such as CNN-SVM^37^ (98.38%), CNN^25^ (92%), and the hybrid CNN-Random Forest model^8^ (91%). Although the CNN-LSTM^51^ model reported a competitive accuracy of 98.17% on a different dataset, our results demonstrate that the proposed model offers superior performance and robustness for the considered dataset.

Class weights were applied to address dataset imbalances, particularly improving classification for underrepresented diseases like Bacterial Blight. Moreover, both DenseNet121 and EfficientNetV2B0 demonstrated scalability, showing potential for application across other crops with minimal retraining, addressing a key limitation in agricultural AI systems. In conclusion, DenseNet121 demonstrates superior performance across all key metrics, offering a reliable path to more accurate, efficient, and sustainable disease detection in agriculture.

### Severity estimation performance

**Fig. 12:**
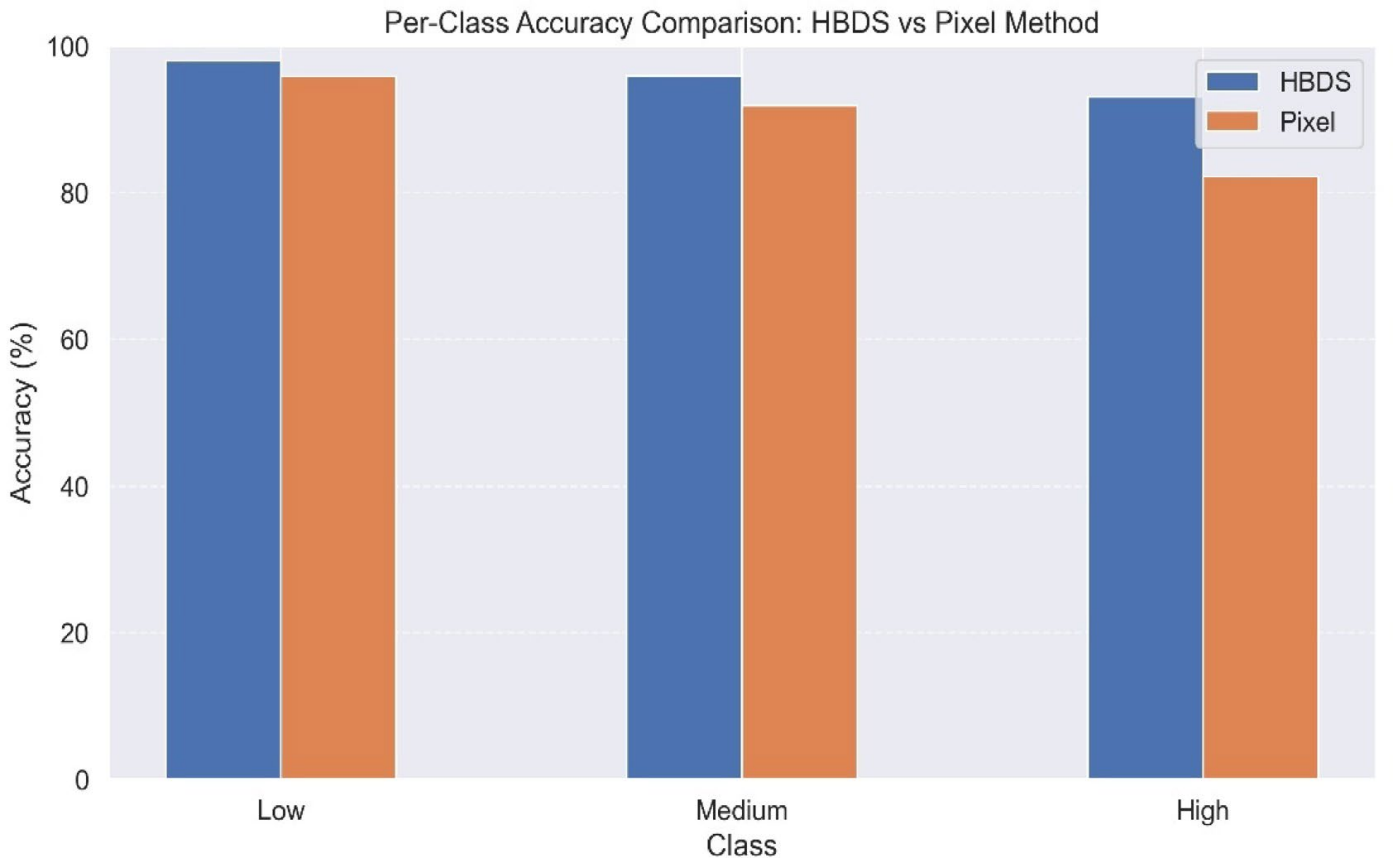
Per-class accuracy comparison between HBDS and Pixel-based Severity Estimation.

The performance of the proposed Healthy-Based Deviation Scoring (HBDS) method for severity classification was evaluated across three severity levels: Low, Medium, and High. The HBDS approach integrates Grad-CAM ++ for lesion region localization, Mahalanobis distance-based deviation scoring, and Gaussian Mixture Model (GMM) clustering. This framework avoids manually predefined pixel thresholds and instead learns a distributional deviation from healthy baselines, addressing the limitations posed by rigid pixel-based approaches. To assess its performance, HBDS was compared against a traditional pixel percentage method that computes lesion severity based on binary thresholding. Figure 12 illustrates the per-class accuracy achieved by both methods. HBDS consistently outperformed the pixel-based method across all three severity classes, achieving 97.4%, 96.1%, and 93.8% for Low, Medium, and High severity levels, respectively. In contrast, the pixel method underperformed in the high-severity class, with only 82.4% accuracy, demonstrating its tendency to misclassify dense lesion regions due to over- or under-segmentation.

**Fig. 13:**
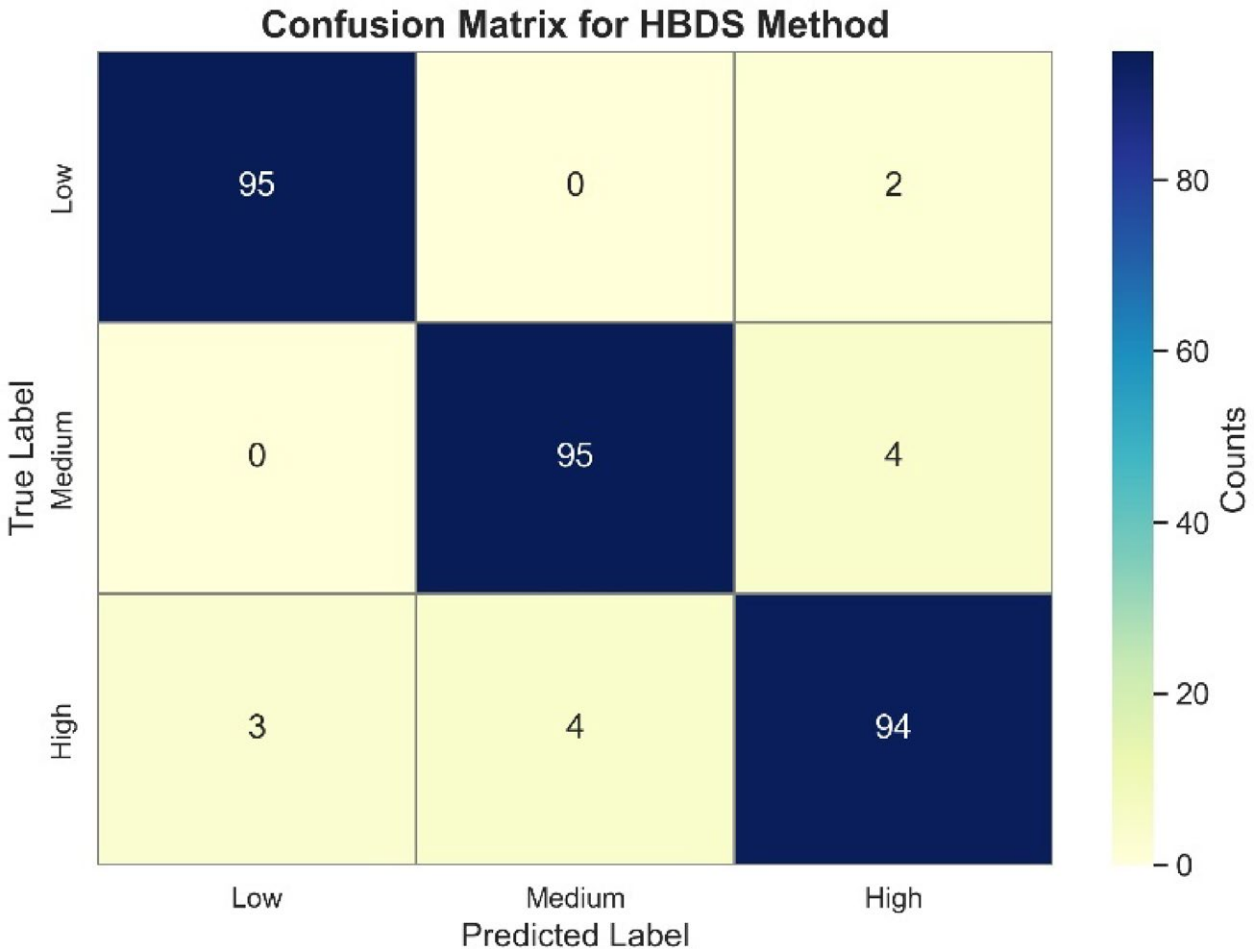
Confusion matrix for severity prediction using HBDS method.

The confusion matrix of HBDS severity predictions, shown in Fig. 13, further highlights the model’s reliability. Out of 100 samples per class, the model correctly predicted 95 Low, 95 Medium and 94 High severity samples. Misclassifications were minimal and primarily observed between adjacent severity levels (e.g., High vs Medium), which is expected in subjective severity annotations. To further validate the reliability of the HBDS model, its predictions have been compared against expert-assigned severity labels (manual annotation). This comparison was based on 150 manually reviewed samples across severity classes. The severity of pomegranate disease was systematically evaluated by an experienced agricultural expert from a reputed university, ensuring a reliable assessment of infection levels. Following this expert evaluation, the percentage of disease-affected pomegranate fruits was further quantified and investigated in detail to establish the extent of damage and its potential impact on crop quality and yield. The Mean Absolute Error (MAE) was used as a quantitative metric to assess alignment between predicted severity scores and ground truth. MAE is defined as:

12 $$\text{MAE}=\frac{1}{n}\sum_{i=1}^{n}\left|{y}_{i}-{\widehat{y}}_{i}\right|$$

where ${y}_{i}$ is the true label, ${\widehat{y}}_{i}$ is the predicted severity label, and *n* is the total number of samples. Lower MAE values indicate better agreement with expert annotation. As shown in Table 5, HBDS achieved an MAE of 0.061, significantly outperforming the pixel-based method, which yielded 0.121. This indicates that the HBDS framework exhibits greater alignment with expert assessments and is more robust to ambiguous lesion spread patterns.

**Table 5:** Mean absolute error between manual severity labels and model predictions.

Method	Mean absolute error (MAE)
HBDS	0.061
Pixel based	0.121

These results underscore the advantage of learning-based deviation scoring over heuristic thresholding. The HBDS framework demonstrated not only higher accuracy but also better agreement with expert judgment, particularly in distinguishing borderline cases. Previous literature has emphasized the drawbacks of ordinal severity scales and manual scoring in agricultural pathology^49,50^. The HBDS approach effectively addresses these issues by leveraging unsupervised clustering on deviation scores, promoting objective, reproducible severity classification in real-world settings.

### Recommendation module and deployment ıntegration

To bridge the gap between disease identification and actionable treatment, this study incorporates a Recommendation Module built on a Retrieval-Augmented Generation (RAG) framework. Activated after HBDS severity estimation, it takes as input the predicted disease class and severity level (Low, Medium, or High) to generate tailored treatment plans. The knowledge base underpinning this module was rigorously curated from authoritative agricultural journals, government-published guidelines, and standard reference books on pomegranate pathology and management. Each entry was organized with metadata including disease class, severity level, and treatment type (chemical, organic, or biological) and stored in a Qdrant vector database to enable fast and precise similarity-based retrieval. This ensures that only contextually relevant protocols are retrieved, avoiding irrelevant or mismatched treatments.

The retrieved content is processed by a refined Mistral Small 3.1 (24B) domain-constrained LLM, which generates context-aware and scientifically grounded recommendations. By strictly restricting the LLM to pomegranate-related material and retrieved entries, the system minimizes hallucination risk and guarantees factual consistency. Recommendations include both chemical and organic options, dosage levels, and application intervals, providing actionable and flexible treatment alternatives aligned with standard agronomic practices. Reliability was validated through retrieval relevance scoring, which demonstrated strong semantic alignment and expert cross-verification, confirming that the outputs closely matched established protocols. These safeguards ensure that the module delivers severity-aware, accurate, and practically deployable treatment advice.

**Fig. 14:**
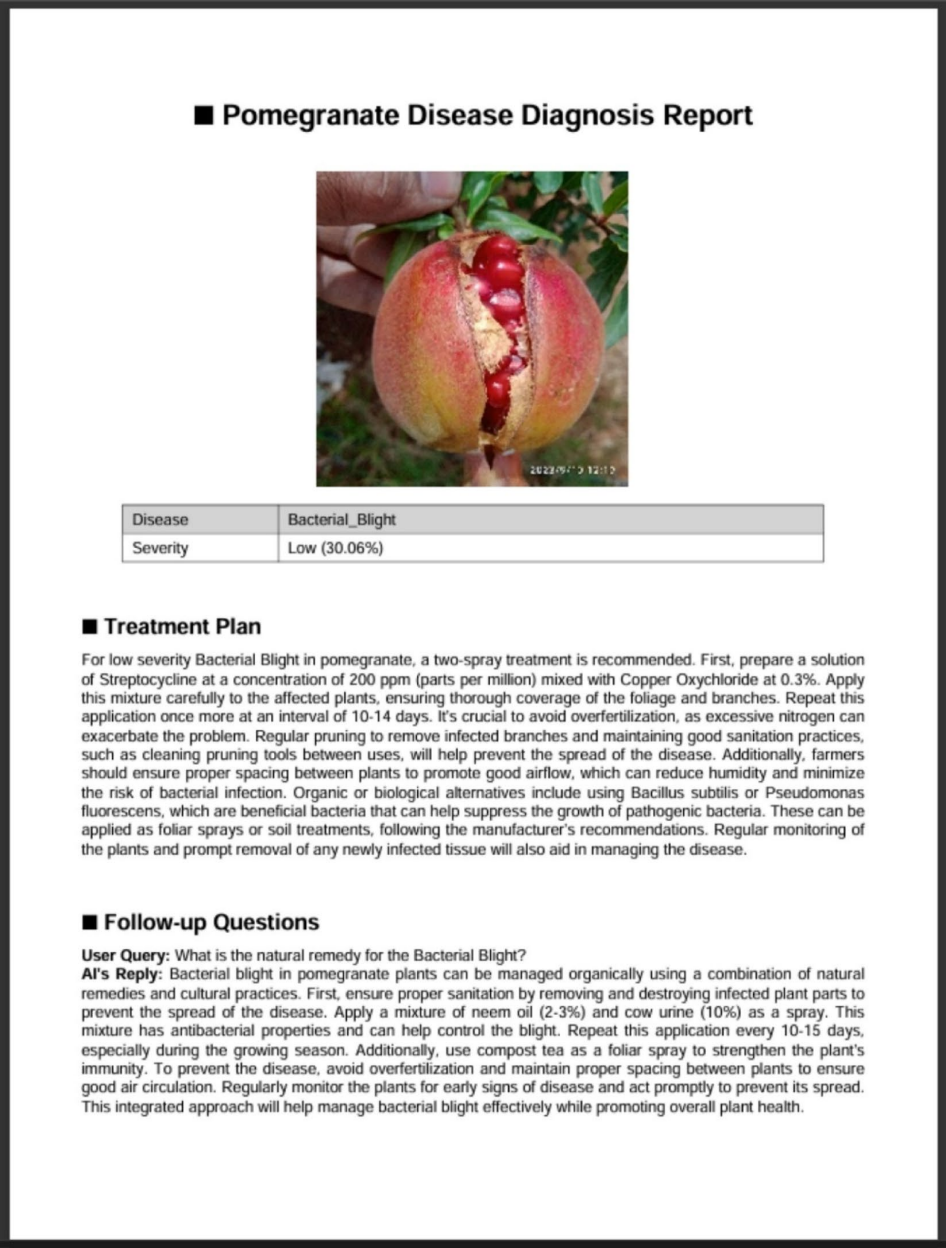
Sample page from the AI-generated disease diagnosis and advisory report. The authors’ system interface generated the report and includes model output, severity classification, and LLM-driven treatment recommendations.

**Fig. 15:**
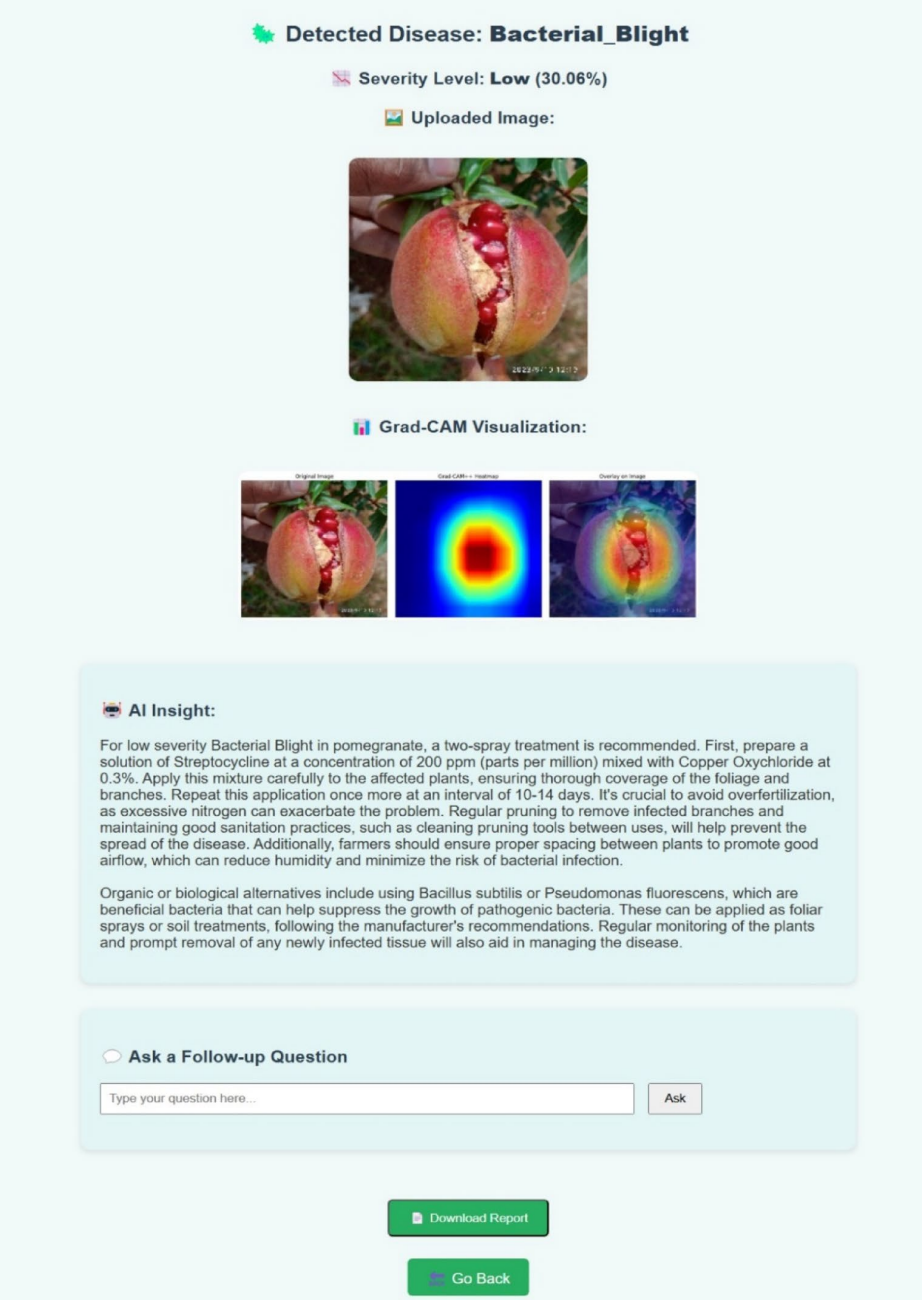
Screenshot of the web-based diagnostic interface developed by the authors, showing image upload, disease prediction, Grad-CAM ++ visualization, and treatment recommendation modules.

An additional feature of the system is automated PDF report generation, which consolidates disease classification results, severity scores, and personalized recommendations into a portable and interpretable summary for farmers and agronomists, as displayed in Fig. 14. This functionality supports flexible adoption by including both chemical and organic suggestions for each severity class. The module is further integrated into a lightweight web interface, enabling users to upload images, view classification and severity predictions, and receive real-time treatment protocols. This design aligns with emerging trends in deployable agricultural decision support systems that integrate multiple AI components within a modular pipeline^45^. A screenshot of this interface is shown in Fig. 15, capturing the user’s journey from prediction to recommendation.

## Conclusion and future work

This study presented an integrated deep learning-based framework for the automatic classification of pomegranate fruit diseases, severity estimation, and treatment recommendation. The classification pipeline utilized six pre-trained convolutional neural networks: DenseNet121, EfficientNetB0V2, MobileNetV2, ResNet50, VGG16, and InceptionV3. These were trained on a domain-annotated dataset comprising 5,099 high-resolution images spanning five disease classes. Among these models, DenseNet121 demonstrated superior performance in terms of classification accuracy and inference efficiency. However, all models were uniformly evaluated under consistent preprocessing and training configurations to ensure a fair comparison. The proposed Health-Based Deviation Scoring (HBDS) approach allowed robust severity estimation, hence surpassing conventional classification. For lesion localization, HBDS combined Grad-CAM ++ with Mahalanobis distance to compute deviation from healthy representations and Gaussian Mixture Models to classify the degrees of severity into Low, Medium, and High. Using comparative evaluation against manual annotations, HBDS showed better alignment with human judgment and produced lower Mean Absolute Error (MAE = 0.061) than pixel-based severity measuring techniques, therefore indicating its relevance for applications in precision agriculture. A retrieval-augmented recommendation system was also incorporated using a domain-specific knowledge base hosted on Qdrant and queried through the Mistral Small 3.1 (24B) language model. The technology produced tailored PDF reports and offered severity-aware therapy recommendations. Designed as a responsive web application, the user interface enhances practical usefulness for end users, including agronomists and farmers, by facilitating real-time image-based diagnosis, interactive chatbot searches, and downloadable treatment summaries.

Future work will focus on optimizing the system for mobile and edge deployments through lightweight model compression and quantization. Additional directions include incorporating multilingual support for broader accessibility, expanding the framework to include lesion segmentation and crop progression monitoring, and adapting the model to other high-value horticultural crops using domain adaptation and transfer learning. These enhancements will further increase the real-world applicability, scalability, and interpretability of the proposed AI-driven agricultural support system.

## Data Availability

Pomegranate Fruit Diseases Dataset for Deep Learning Models ([https://data.mendeley.com/datasets/b6s2rkpmvh/1]) (Mendeley Data).
